# Can social prescribing reach patients most in need? Patterns of (in)equalities in referrals in a representative cohort of older adults in England

**DOI:** 10.1177/17579139251330767

**Published:** 2025-04-28

**Authors:** D Fancourt, A Steptoe

**Affiliations:** Department of Behavioural Science and Health, University College London, 1-19 Torrington Place, London, WC1E 7HB, UK; Department of Behavioural Science and Health, University College London, London, UK

**Keywords:** personalised care, social prescribing, mental health

## Abstract

**Aims:**

Social prescribing (SP) is a mechanism of care referring people to non-clinical forms of support and services in local communities to improve health and wellbeing. But there is much contention over whether SP is provided disproportionately more to individuals who are less disadvantaged. A comprehensive analysis of who is receiving SP from both medical and non-medical referral routes has never been undertaken.

**Methods:**

We used data from 7283 adults aged 50+ in the English Longitudinal Study of Ageing (ELSA), incorporating novel questions on SP into Wave 10. Multiple logistic regression models were used to explore predictors of self-reported referrals to SP.

**Results:**

A total of 495 adults (6.8%) reported receiving an SP referral and 435 (88%) accepted. Referrals were more likely among older adults (odds ratio (OR) = 1.02, confidence interval (CI) = 1.01–1.03), those with chronic pain (OR = 1.78, CI = 1.40–2.27), those who were lonely (OR = 2.20, CI = 1.63–2.97), those from the lowest wealth tertile (OR = 1.59, CI = 1.17–2.18) and those receiving benefits (OR = 2.02, CI = 1.52–2.69). Diagnosed psychiatric conditions and depressive symptoms, sedentary behaviours, cardiovascular conditions, diabetes, and physical inactivity predicted referrals only in minimally adjusted models. But those with multiple long-term conditions were more likely to be referred (OR = 2.02, CI = 1.00–4.08).

**Conclusion:**

There is promising initial evidence that SP referrals are occurring among older adults in England, with high uptake among those referred. Promisingly, those with the highest socio-economic need and most long-term health conditions particularly appear to be receiving support. Mental health appears more of a secondary rather than a primary referral predictor.

## Introduction

Social prescribing (SP) is a mechanism of care referring people to non-clinical forms of support and services in local communities to improve health and wellbeing.^1^ These forms of support and services can include many different activities, although in the UK, the National Academy for Social Prescribing describes them under five main categories: arts and culture, heritage, natural environment, physical activity, and advice and information.

While initially traced back to initiatives developed in the UK, SP is now gaining global traction, with many countries implementing programmes to address social determinants of health, with the aim of reducing health inequalities.^2^ However, this claim has proved controversial, with concerns that SP cannot address upstream determinants of inequalities and could even be provided disproportionately more to individuals who are less disadvantaged.^3–5^ While SP is an individual-level intervention, so does not address structural societal drivers of inequalities, it does broaden existing health service provision and could increase the uptake of community activities among those least likely to engage, as well as supporting self-management of existing mental and physical health conditions.^6^ The challenge is that few studies have analysed issues of uptake and equity.

In the UK, SP is included in the National Health Service (NHS) Long-Term Plan for ‘tackling health inequalities’, with the specific aims of supporting people with mental health problems, long-term conditions, loneliness/isolation, and complex social needs. Its national roll-out as a programme across England provides a fertile opportunity to explore equity of access and success in reaching these populations. However, it is a major challenge to assess who is receiving SP. Local evaluations can be too small in scale, while electronic patient records often contain inconsistent coding of SP referrals, poor recording of wider determinants of health, and no details of referrals from sources other than GPs, leading to conclusions that they cannot be used to assess equity of referrals.^7^ Thus, exploring SP referral patterns in wider data sources is crucial to understanding if SP is truly reaching individuals most in need.

In 2020, we succeeded in incorporating bespoke questions on SP into a nationally representative cohort study of richly phenotyped adults aged 50+ living in England. This is the first report on the first wave of these data. Specifically, we sought to identify patterns of SP referrals and their uptake based on mental, physical, social, socioeconomic and behavioural factors and explore if SP is reaching those the NHS is aiming to reach: a vital question if SP is to play a role in addressing health and social inequalities.

## Methods

We used data from Waves 9 and 10 of the English Longitudinal Study of Ageing, a multiwave, nationally representative cohort study of community dwelling adults aged 50 years and older.^8^ Questions on SP in Wave 10 (2021/23) asked ‘In the past 2 years, has a doctor, social worker or other health professional referred you to take part in any of the following: (i) arts, crafts, music, reading groups, or social groups, (ii) gardening or nature activities, (iii) outdoor health or fitness activities/clubs, (iv) indoor exercise or other clubs/activities, (v) adult learning or skills development training, (vi) employment or benefit support, or (vii) other social, community or volunteering activity’. Responses were ‘No’, ‘Yes, but I did not accept’, ‘Yes, I attended just one session’, or ‘Yes, I attended more than one session’. A total of 7283 adults aged 50+ answered the self-completion questionnaire at wave 10 so were included in analyses.

As this reporting on SP focused on experiences ‘in the past 2 years’, we explored how SP was patterned according to social and health inequalities by using data from the beginning of that 2-year period, that is, Wave 9 of ELSA (2018/19; just prior to any reported engagement in SP). Basic demographic predictors included age (years) and sex. Mental predictors included diagnosed psychiatric condition and depressive symptoms (⩾3 on the eight-item Center for Epidemiologic Studies Depression Scale; CES-D). Physical predictors included diagnoses of cardiovascular conditions (hypertension, angina, heart attack, heart failure, heart murmur, abnormal heart rhythm, stroke, or heart disease), diabetes, respiratory conditions (lung disease or asthma), musculoskeletal conditions (arthritis or osteoporosis), dementia, or cancer. We also created a count variable of the number of conditions to assess multimorbidity and measured chronic pain. Social predictors included the three-item UCLA loneliness scale (⩾6 indicating loneliness), marital status (married/cohabiting versus other), and an index of whether people had weekly social interaction with friends or family or not. Socio-economic predictors included net non-pension wealth (tertiles), receipt of any state benefits, area deprivation (from linking lower layer super output area data to Index of Multiple Deprivation (IMD) from the 2021 census), retirement status, educational attainment (no qualifications/basic vocational qualifications versus General Certificate of Education (GCE)/ Ordinary level (O level) versus Advanced level (A level) versus degree), and urban living (yes versus no). Behavioural predictors included sedentary behaviour (physical activity less than once a week), alcohol consumption (⩾5 times a week), and current smoking.

Multiple logistic regression models were used to explore predictors of receiving an SP referral. Multiple imputation by chained equations was used to provide 30 imputed data sets, incorporating all predictors and identified auxiliary variables relating to social behaviours (see supplementary Table 2 for patterns of missingness). Main analyses binarised SP as receiving an SP referral (regardless of uptake) and sensitivity analyses instead explored uptake versus not/no referral. Model 1 was adjusted for age, sex, educational attainment, and urbanicity; model 2 = model 1 + each category of predictors detailed above in turn; model 3 = model 1 + all categories of predictors together; and model 4 = model 1 + all categories of predictors, using a count of chronic conditions instead of individual binary indicators.

## Results

**Figure 1:**
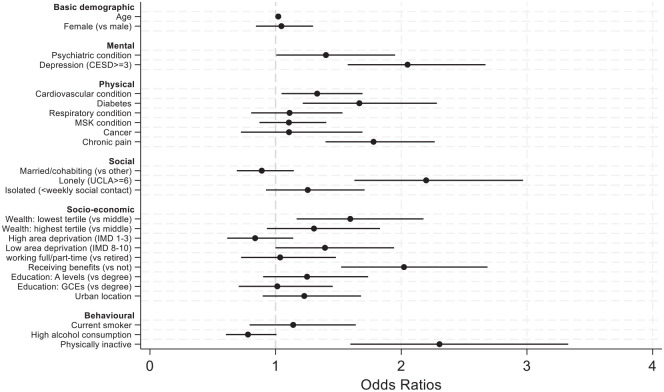
Predictors of SP referrals in logistic regression models (model 2)

A total of 495 adults (6.8%) reported receiving an SP referral, and 435 (88%) accepted. Exercise classes were the most frequent intervention, followed by arts groups, nature-based activities, and adult learning. Of those referred to SP, 56% were female, average age 67.1 (SE = 0.5 years) (see Supplementary Tables 1–2). Results from model 2 are reported in Figure 1. Full tables of results (including subsequent models) are in Supplementary Table 3.

Gender did not predict referrals, although referrals increased with age (odds ratio (OR) = 1.02, confidence interval (CI) = 1.01–1.03). Those with diagnosed psychiatric conditions and above-threshold depressive symptoms were more likely to be referred (OR = 1.40, CI = 1.01–1.95 and OR = 1.99, CI = 1.52–2.60). Referrals were also more common for individuals with cardiovascular conditions (OR = 1.33, CI = 1.05–1.69), diabetes (OR = 1.67, CI = 1.22–2.28), and chronic pain (OR = 1.78, CI = 1.40–2.27). Those who were lonely were more than twice as likely to be referred (OR = 2.20, CI = 1.63–2.97), although marital status and frequency of social contact were not related. People from the lowest wealth tertile were also most likely to receive referrals (OR = 1.59, CI = 1.17–2.18), as were individuals receiving benefits (OR = 2.02, CI = 1.52–2.69). But there was no association with IMD score, educational attainment, urban dwelling, or working status. Behaviourally, those who were physically inactive were most likely to receive referrals (OR = 2.30, CI = 1.59–3.33), but smoking and alcohol were not related.

Results were all maintained in model 3, apart from mental health measures, physical inactivity, and cardiovascular conditions and diabetes (see Supplementary Table 3). However, when using a count of chronic conditions, those with 4+ chronic conditions were twice as likely to be referred (OR = 2.02, CI = 1.00–4.08). Sensitivity analyses focusing on the uptake of SP (rather than just referral) produced very similar findings (see Supplementary Table 4).

## Discussion

SP referrals are evidently occurring among older adults in England as part of the national SP programme. Uptake of reported referrals was higher than expected: a large study that, like this study, explored referrals from medical and non-medical pathways reported 90% of referrals leading to contact with a link worker, but only 38% leading to an intervention.^9^ However, this study focused exclusively on older adults, who may have had higher acceptance rates, or recall bias may have meant that those who declined referrals were less likely to remember having received them. Furthermore, this study asked specifically about seven types of community SP activities that people can be referred to, based around the National Academy for Social Prescribing (NASP) pillars of SP. However, health and social care professionals can also refer people to broader types of non-clinical support such as support with housing, food security, and transportation needs that were not examined in this study. Therefore, these results are not intended to reflect referral prevalence for all non-clinical services.

Promisingly, there were clear indications that SP is reaching some of the target populations it is designed to reach, at least among older adults who have been referred. In particular, individuals who are lonely, have multiple chronic conditions, and who are at the most risk of social inequalities had the highest odds of reporting referrals, which is in line with NHS aims to target these groups specifically. Prior research has shown these groups as the least likely to engage in community-based activities like those offered by social prescribing, as well as the most likely to experience poor long-term health.^10^ This suggests that SP does have the potential for reaching individuals who are more disadvantaged. However, there was some slight indication that individuals in the least deprived areas may be slightly more likely to receive referrals, which could be indicative of inconsistencies in the geographical availability of SP.^11^ Notably, the largest predictors of referrals were loneliness and physical inactivity, which are identified risk factors for mental and physical illness. Loneliness was the predictor that was most strongly maintained once accounting for other predictors. Also of note is that mental health became a less strong predictor once taking these other needs into account, suggesting mental health is more of a secondary referral reason. However, further research is needed to explore how initial uptake of referrals translates to subsequent engagement patterns and, in particular, whether inequalities shape participants’ capacity to benefit from the interventions provided.

This is the first wave of ELSA to include questions on SP, so these analyses present a preliminary exposition of the data. The small sample size reporting a referral precluded specific analyses for each activity subtype or interaction analyses to explore intersectionality. Data gathered in future waves will allow more sophisticated longitudinal analyses also focusing on outcomes. As the sample size of those reporting SP referrals grows (which is anticipated given the ongoing expansion of the national SP programme), additional analyses in future waves will also be possible, exploring issues such as intersectionality and potential curvilinear patterns within predictor variables. In addition, this study did not attempt to explore whether referrals provided were well targeted or met the needs of patients. However, overall, these data from a representative cohort study give indications of SP from all referrers (not just GPs), providing important data to triangulate with SP data from electronic patient records. As SP programmes grow in scale internationally, the inclusion of similar measures of SP referrals in other longitudinal cohort studies is recommended.

## Supplemental Material

sj-docx-1-rsh-10.1177_17579139251330767 – Supplemental material for Can social prescribing reach patients most in need? Patterns of (in)equalities in referrals in a representative cohort of older adults in England Supplemental material, sj-docx-1-rsh-10.1177_17579139251330767 for Can social prescribing reach patients most in need? Patterns of (in)equalities in referrals in a representative cohort of older adults in England by D Fancourt and A Steptoe in Perspectives in Public Health
