# Supplementary material for: Can social prescribing reach patients most in need? Patterns of (in)equalities in referrals in a representative cohort of older adults in England
Source: Perspect Public Health. 2025 Apr 28;145(4):198–201. doi: 10.1177/17579139251330767 (PMC12322337; doi:10.1177/17579139251330767)
Supplement: sj-docx-1-rsh-10.1177_17579139251330767 – Supplemental material for Can social prescribing reach patients most in need? Patterns of (in)equalities in referrals in a representative cohort of older adults in England [file sj-docx-1-rsh-10.1177_17579139251330767.docx]

# Supplementary Tables

| Supplementary Table 1: Descriptive statistics by SP (referral); proportions and confidence intervals | | | | | |
| --- | --- | --- | --- | --- | --- |
|  | Not referred to SP |  | Referred to SP |  |  |
| Age (years), mean (SE) | 65.3 (0.1) |  | 67.1 (0.5) |  |  |
| Sex |  |  |  |  |  |
| Male | 0.45 [0.44, 0.46] |  | 0.44 [0.39, 0.49] |  |  |
| Female | 0.55 [0.54, 0.56] |  | 0.56 [0.51, 0.61] |  |  |
| Educational attainment |  |  |  |  |  |
| Degree | 0.28 [0.27, 0.29] |  | 0.21 [0.16, 0.26] |  |  |
| nvq3 A level/higher education | 0.34 [0.33, 0.36] |  | 0.38 [0.33, 0.43] |  |  |
| nvq2/gce o level | 0.38 [0.37, 0.39] |  | 0.41 [0.35, 0.46] |  |  |
| Urban dwelling |  |  |  |  |  |
| No | 0.27 [0.26, 0.28] |  | 0.21 [0.16, 0.25] |  |  |
| Yes | 0.73 [0.72, 0.74] |  | 0.79 [0.75, 0.84] |  |  |
| Diagnosed psychiatric condition |  |  |  |  |  |
| No | 0.92 [0.92, 0.93] |  | 0.88 [0.85, 0.91] |  |  |
| Yes | 0.08 [0.07, 0.08] |  | 0.12 [0.09, 0.15] |  |  |
| Depression (CESD>=3) |  |  |  |  |  |
| No | 0.82 [0.81, 0.83] |  | 0.69 [0.63, 0.74] |  |  |
| Yes | 0.18 [0.17, 0.19] |  | 0.31 [0.26, 0.37] |  |  |
| Diagnosed cardiovascular condition |  |  |  |  |  |
| No | 0.65 [0.64, 0.67] |  | 0.53 [0.47, 0.58] |  |  |
| Yes | 0.35 [0.33, 0.36] |  | 0.47 [0.42, 0.53] |  |  |
| Diagnosed diabetes |  |  |  |  |  |
| No | 0.92 [0.91, 0.92] |  | 0.84 [0.80, 0.88] |  |  |
| Yes | 0.08 [0.08, 0.09] |  | 0.16 [0.12, 0.20] |  |  |
| Diagnosed lung condition |  |  |  |  |  |
| No | 0.87 [0.87, 0.88] |  | 0.84 [0.80, 0.88] |  |  |
| Yes | 0.13 [0.12, 0.13] |  | 0.16 [0.12, 0.20] |  |  |
| Diagnosed MSK condition |  |  |  |  |  |
| No | 0.66 [0.65, 0.68] |  | 0.56 [0.51, 0.61] |  |  |
| Yes | 0.34 [0.32, 0.35] |  | 0.44 [0.39, 0.49] |  |  |
| Diagnosed cancer |  |  |  |  |  |
| No | 0.94 [0.93, 0.94] |  | 0.92 [0.89, 0.95] |  |  |
| Yes | 0.06 [0.06, 0.07] |  | 0.08 [0.05, 0.11] |  |  |
| Frequent/chronic pain |  |  |  |  |  |
| No | 0.61 [0.60, 0.62] |  | 0.44 [0.39, 0.49] |  |  |
| Yes | 0.39 [0.38, 0.40] |  | 0.56 [0.51, 0.61] |  |  |
| Marital status |  |  |  |  |  |
| Not married/cohabiting | 0.25 [0.24, 0.26] |  | 0.33 [0.28, 0.37] |  |  |
| Married/cohabiting | 0.75 [0.74, 0.76] |  | 0.67 [0.63, 0.72] |  |  |
| Lonely |  |  |  |  |  |
| No | 0.81 [0.80, 0.83] |  | 0.66 [0.60, 0.72] |  |  |
| Yes | 0.19 [0.17, 0.20] |  | 0.34 [0.28, 0.40] |  |  |
| Social contact |  |  |  |  |  |
| Weekly or more | 0.67 [0.65, 0.69] |  | 0.62 [0.55, 0.68] |  |  |
| Less than weekly | 0.33 [0.31, 0.35] |  | 0.38 [0.32, 0.45] |  |  |
| Net non-pension wealth (tertiles) |  |  |  |  |  |
| 1 - lowest wealth quintile | 0.35 [0.33, 0.36] |  | 0.50 [0.44, 0.55] |  |  |
| 3 - highest wealth quintile | 0.32 [0.31, 0.33] |  | 0.27 [0.22, 0.32] |  |  |
| Index of multiple deprivation |  |  |  |  |  |
| IMD score 1-3 | 0.40 [0.39, 0.42] |  | 0.31 [0.25, 0.36] |  |  |
| IMD score 8-10 | 0.18 [0.17, 0.19] |  | 0.29 [0.24, 0.35] |  |  |
| Working status |  |  |  |  |  |
| Not working | 0.60 [0.58, 0.61] |  | 0.68 [0.62, 0.73] |  |  |
| Working full/part-time | 0.40 [0.39, 0.42] |  | 0.32 [0.27, 0.38] |  |  |
| Receiving benefits |  |  |  |  |  |
| No | 0.86 [0.85, 0.87] |  | 0.71 [0.67, 0.76] |  |  |
| Yes | 0.14 [0.13, 0.15] |  | 0.29 [0.24, 0.33] |  |  |
| Current smoker |  |  |  |  |  |
| No | 0.90 [0.89, 0.90] |  | 0.89 [0.85, 0.92] |  |  |
| Yes | 0.10 [0.10, 0.11] |  | 0.11 [0.08, 0.15] |  |  |
| Alcohol consumption |  |  |  |  |  |
| <5 times a week | 0.77 [0.76, 0.78] |  | 0.82 [0.78, 0.86] |  |  |
| 5+ times a week | 0.23 [0.22, 0.24] |  | 0.18 [0.14, 0.22] |  |  |
| Physically inactive |  |  |  |  |  |
| No | 0.94 [0.94, 0.95] |  | 0.87 [0.83, 0.91] |  |  |
| Yes | 0.06 [0.05, 0.06] |  | 0.13 [0.09, 0.17] |  |  |
| Number of observations | . |  | . |  |  |
| *N for SP=486, N for control=6,797* | | | | |  |

| Supplementary Table 2: Descriptive statistics for the sample showing sample size and missingness | | | |
| --- | --- | --- | --- |
| Variable | Sample size (%) |  |  |
| N | 7,283 |  |  |
| Sex |  |  |  |
| Male | 3,289 (45.2%) |  |  |
| Female | 3,994 (54.8%) |  |  |
| Educational attainment |  |  |  |
| Degree | 1,405 (19.3%) |  |  |
| nvq3 A level/higher education | 1,824 (25.0%) |  |  |
| nvq2/gce o level | 2,038 (28.0%) |  |  |
| Missing | 2,016 (27.7%) |  |  |
| Urban dwelling |  |  |  |
| No | 1,455 (20.0%) |  |  |
| Yes | 3,869 (53.1%) |  |  |
| Missing | 1,959 (26.9%) |  |  |
| Diagnosed psychiatric condition |  |  |  |
| No | 6,693 (91.9%) |  |  |
| Yes | 585 (8.0%) |  |  |
| Missing | 5 (0.1%) |  |  |
| Depression (CESD>=3) |  |  |  |
| No | 5,609 (77.0%) |  |  |
| Yes | 1,308 (18.0%) |  |  |
| Missing | 366 (5.0%) |  |  |
| Diagnosed cardiovascular condition |  |  |  |
| No | 4,698 (64.5%) |  |  |
| Yes | 2,584 (35.5%) |  |  |
| Missing | 1 (0.0%) |  |  |
| Diagnosed diabetes |  |  |  |
| No | 6,631 (91.0%) |  |  |
| Yes | 649 (8.9%) |  |  |
| Missing | 3 (0.0%) |  |  |
| Diagnosed lung condition |  |  |  |
| No | 6,346 (87.1%) |  |  |
| Yes | 933 (12.8%) |  |  |
| Missing | 4 (0.1%) |  |  |
| Diagnosed MSK condition |  |  |  |
| No | 4,784 (65.7%) |  |  |
| Yes | 2,495 (34.3%) |  |  |
| Missing | 4 (0.1%) |  |  |
| Diagnosed cancer |  |  |  |
| No | 6,808 (93.5%) |  |  |
| Yes | 473 (6.5%) |  |  |
| Missing | 2 (0.0%) |  |  |
| Frequent/chronic pain |  |  |  |
| No | 4,152 (57.0%) |  |  |
| Yes | 2,808 (38.6%) |  |  |
| Missing | 323 (4.4%) |  |  |
| Marital status |  |  |  |
| Not married/cohabiting | 1,876 (25.8%) |  |  |
| Married/cohabiting | 5,407 (74.2%) |  |  |
| Lonely |  |  |  |
| No | 3,972 (54.5%) |  |  |
| Yes | 843 (11.6%) |  |  |
| Missing | 2,468 (33.9%) |  |  |
| Social contact |  |  |  |
| Weekly or more | 3,392 (46.6%) |  |  |
| Less than weekly | 1,621 (22.3%) |  |  |
| Missing | 2,270 (31.2%) |  |  |
| Net non-pension wealth (tertiles) |  |  |  |
| 1 - lowest wealth quintile | 1,770 (24.3%) |  |  |
| 2 | 1,766 (24.2%) |  |  |
| 3 - highest wealth quintile | 1,766 (24.2%) |  |  |
| Missing | 1,981 (27.2%) |  |  |
| Index of multiple deprivation |  |  |  |
| 1 | 2,188 (30.0%) |  |  |
| 2 | 2,228 (30.6%) |  |  |
| 3 | 2,867 (39.4%) |  |  |
| Working status |  |  |  |
| Not working | 3,507 (48.2%) |  |  |
| Working full/part-time | 1,758 (24.1%) |  |  |
| Missing | 2,018 (27.7%) |  |  |
| Receiving benefits |  |  |  |
| No | 6,133 (84.2%) |  |  |
| Yes | 1,068 (14.7%) |  |  |
| Missing | 82 (1.1%) |  |  |
| Current smoker |  |  |  |
| No | 5,794 (79.6%) |  |  |
| Yes | 642 (8.8%) |  |  |
| Missing | 847 (11.6%) |  |  |
| Alcohol consumption |  |  |  |
| <5 times a week | 5,585 (76.7%) |  |  |
| 5+ times a week | 1,630 (22.4%) |  |  |
| Missing | 68 (0.9%) |  |  |
| Physically inactive |  |  |  |
| No | 6,823 (93.7%) |  |  |
| Yes | 460 (6.3%) |  |  |

Supplementary Table 3: Logistic Regression Model for SP (referrals to SP); odds ratios and confidence intervals

|  |  |  | 1 | | 2 | | | 3 | |
| --- | --- | --- | --- | --- | --- | --- | --- | --- | --- |
| Age (years) | | | 1.02 [1.01, 1.03] | *** | | 1.02 [1.01, 1.04] | *** | 1.02 [1.01, 1.04] | ** |
| Sex | Female | | 1.05 [0.84, 1.30] |  | | 0.96 [0.78, 1.20] |  | 1.00 [0.80, 1.27] |  |
| Diagnosed psychiatric condition | Yes | |  |  | | 1.40 [1.01, 1.95] | * | 1.08 [0.75, 1.55] |  |
| Depression (CESD>=3) | Yes | |  |  | | 2.05 [1.57, 2.67] | *** | 1.19 [0.85, 1.66] |  |
| Diagnosed cardiovascular condition | Yes | |  |  | | 1.33 [1.05, 1.69] | * | 1.24 [0.97, 1.58] |  |
| Diagnosed diabetes | Yes | |  |  | | 1.67 [1.22, 2.28] | ** | 1.37 [0.98, 1.91] |  |
| Diagnosed lung condition | Yes | |  |  | | 1.11 [0.81, 1.53] |  | 0.96 [0.69, 1.35] |  |
| Diagnosed MSK condition | Yes | |  |  | | 1.11 [0.87, 1.40] |  | 1.01 [0.79, 1.30] |  |
| Diagnosed cancer | Yes | |  |  | | 1.11 [0.72, 1.69] |  | 1.05 [0.68, 1.63] |  |
| Frequent/chronic pain | Yes | |  |  | | 1.78 [1.40, 2.27] | *** | 1.44 [1.11, 1.87] | ** |
| Marital status | Married/cohabiting | |  |  | | 0.89 [0.69, 1.14] |  | 1.02 [0.78, 1.33] |  |
| Lonely | Yes | |  |  | | 2.20 [1.63, 2.97] | *** | 1.70 [1.21, 2.39] | ** |
| Social contact | Less than weekly | |  |  | | 1.26 [0.92, 1.71] |  | 1.18 [0.86, 1.64] |  |
| Net non-pension wealth (tertiles) | 1 - lowest wealth quintile | |  |  | | 1.59 [1.17, 2.18] | ** | 1.43 [1.03, 1.99] | * |
|  | 3 - highest wealth quintile | |  |  | | 1.31 [0.93, 1.83] |  | 1.41 [1.00, 2.00] | * |
| Index of multiple deprivation | IMD score 1-3 | |  |  | | 0.84 [0.61, 1.14] |  | 0.83 [0.61, 1.13] |  |
|  | IMD score 8-10 | |  |  | | 1.39 [1.00, 1.94] |  | 1.31 [0.94, 1.83] |  |
| Working status | Working full/part-time | |  |  | | 1.04 [0.73, 1.48] |  | 1.15 [0.81, 1.64] |  |
| Receiving benefits | Yes | |  |  | | 2.02 [1.52, 2.69] | *** | 1.50 [1.10, 2.04] | * |
| Educational attainment | nvq3 A level/higher education | |  |  | | 1.25 [0.90, 1.74] |  | 1.22 [0.87, 1.70] |  |
|  | nvq2/gce o level | |  |  | | 1.01 [0.71, 1.45] |  | 0.97 [0.67, 1.40] |  |
| Urban dwelling | Yes | |  |  | | 1.23 [0.90, 1.68] |  | 1.21 [0.88, 1.66] |  |
| Current smoker | Yes | |  |  | | 1.14 [0.79, 1.64] |  | 0.88 [0.60, 1.31] |  |
| Alcohol consumption | 5+ times a week | |  |  | | 0.78 [0.60, 1.01] |  | 0.84 [0.65, 1.09] |  |
| Physically inactive | Yes | |  |  | | 2.30 [1.59, 3.33] | *** | 1.28 [0.84, 1.93] |  |
| Intercept | | | 0.02 [0.01, 0.04] | *** | |  |  | 0.00 [0.00, 0.02] | *** |
| Number of observations | | | 7283 |  | | 7283 |  | 7283 |  |

**** p<.001, ** p<.01, * p<.05 N=7,283*

*Column 1: adjusted for age and sex; Column 2: adjusted for model 1 + each category in turn (model 1 not shown as coefficients varied depending on which additional predictors were included); Column 3: adjusted for model 1 + each category altogether;*

*Reference categories: Educational attainment - degree; wealth - mid quintile*

Supplementary Table 4: Logistic Regression Model for SP (uptake of SP); odds ratios and confidence intervals

|  | | |  |  | | 1 | | | 2 | | | 3 | | |
| --- | --- | --- | --- | --- | --- | --- | --- | --- | --- | --- | --- | --- | --- | --- |
| Age (years) | | | | 1.02 [1.01, 1.04] | | *** | 1.02 [1.01, 1.04] | | *** | 1.02 [1.01, 1.04] | | ** |  |  |
| Sex | Female | | | 1.05 [0.83, 1.32] | |  | 0.98 [0.77, 1.24] | |  | 1.01 [0.79, 1.30] | |  |  |  |
| Diagnosed psychiatric condition | Yes | | |  | |  | 1.32 [0.91, 1.92] | |  | 1.01 [0.68, 1.51] | |  |  |  |
| Depression (CESD>=3) | Yes | | |  | |  | 1.85 [1.41, 2.42] | | *** | 1.11 [0.78, 1.56] | |  |  |  |
| Diagnosed cardiovascular condition | Yes | | |  | |  | 1.28 [0.99, 1.66] | |  | 1.21 [0.93, 1.57] | |  |  |  |
| Diagnosed diabetes | Yes | | |  | |  | 1.74 [1.25, 2.42] | | ** | 1.47 [1.04, 2.08] | | * |  |  |
| Diagnosed lung condition | Yes | | |  | |  | 1.10 [0.79, 1.53] | |  | 0.97 [0.69, 1.37] | |  |  |  |
| Diagnosed MSK condition | Yes | | |  | |  | 1.08 [0.84, 1.38] | |  | 1.00 [0.77, 1.29] | |  |  |  |
| Diagnosed cancer | Yes | | |  | |  | 1.10 [0.71, 1.70] | |  | 1.05 [0.67, 1.64] | |  |  |  |
| Frequent/chronic pain | Yes | | |  | |  | 1.74 [1.35, 2.25] | | *** | 1.47 [1.11, 1.93] | | ** |  |  |
| Marital status | Married/cohabiting | | |  | |  | 0.85 [0.64, 1.13] | |  | 0.94 [0.70, 1.26] | |  |  |  |
| Lonely | Yes | | |  | |  | 1.90 [1.32, 2.73] | | *** | 1.52 [1.01, 2.29] | | * |  |  |
| Social contact | Less than weekly | | |  | |  | 1.32 [0.96, 1.81] | |  | 1.26 [0.90, 1.76] | |  |  |  |
| Net non-pension wealth (tertiles) | 1 - lowest wealth quintile | | |  | |  | 1.47 [1.03, 2.11] | | * | 1.32 [0.91, 1.92] | |  |  |  |
|  | 3 - highest wealth quintile | | |  | |  | 1.34 [0.91, 1.96] | |  | 1.45 [0.98, 2.13] | |  |  |  |
| Index of multiple deprivation | IMD score 1-3 | | |  | |  | 0.78 [0.57, 1.07] | |  | 0.78 [0.57, 1.06] | |  |  |  |
|  | IMD score 8-10 | | |  | |  | 1.39 [0.96, 2.01] | |  | 1.30 [0.89, 1.90] | |  |  |  |
| Working status | Working full/part-time | | |  | |  | 0.95 [0.69, 1.33] | |  | 1.03 [0.74, 1.44] | |  |  |  |
| Receiving benefits | Yes | | |  | |  | 1.97 [1.47, 2.65] | | *** | 1.52 [1.10, 2.10] | | * |  |  |
| Educational attainment | nvq3 A level/higher education | | |  | |  | 1.23 [0.88, 1.72] | |  | 1.19 [0.85, 1.68] | |  |  |  |
|  | nvq2/gce o level | | |  | |  | 0.99 [0.68, 1.45] | |  | 0.95 [0.65, 1.40] | |  |  |  |
| Urban dwelling | Yes | | |  | |  | 1.15 [0.82, 1.61] | |  | 1.14 [0.81, 1.60] | |  |  |  |
| Current smoker | Yes | | |  | |  | 1.22 [0.82, 1.82] | |  | 0.96 [0.62, 1.49] | |  |  |  |
| Alcohol consumption | 5+ times a week | | |  | |  | 0.80 [0.62, 1.05] | |  | 0.87 [0.67, 1.14] | |  |  |  |
| Physically inactive | Yes | | |  | |  | 2.03 [1.37, 3.01] | | *** | 1.14 [0.74, 1.76] | |  |  |  |
| Intercept | | | | 0.01 [0.01, 0.03] | | *** |  | |  | 0.01 [0.00, 0.02] | | *** |  |  |
| Number of observations | | | | 7283 | |  | 7283 | |  | 7283 | |  |  |  |

**** p<.001, ** p<.01, * p<.05 N=7,283*

*Column 1: adjusted for age and sex; Column 2: adjusted for model 1 + each category in turn (model 1 not shown as coefficients varied depending on which additional predictors were included); Column 3: adjusted for model 1 + each category altogether;*

*Reference categories: Educational attainment - degree; wealth - mid quintile*
